# Breeding objectives and trait preferences of Nguni goat farmers in Limpopo province, South Africa

**DOI:** 10.1007/s11250-025-04281-2

**Published:** 2025-02-05

**Authors:** Madumetja Cyril Mathapo, Thobela Louis Tyasi, Thinawanga Joseph Mugwabana

**Affiliations:** School of Agriculture and Environmental Sciences, Department of Agricultural Economics and Animal Production, University of Limpopo, Private Bag X1106, Sovenga 0727, Limpopo, South Africa

**Keywords:** Breeding practices, Community-based breeding program, Nguni goats, Production objectives, Trait preferences

## Abstract

Understanding the farmers production objectives, breeding practices and trait preferences is important in designing and implementing Community-based breeding program (CBBP) for genetic improvement of livestock. Thus, this study assessed production objectives, breeding practices and trait preferences of Nguni goat farmers in four agroecological zones of Limpopo Province, South Africa. Using a participatory approach, a semi-structured questionnaire was administered to 33 Nguni goat farmers. Frequencies, percentages, chi-square test, and Kruskal–Wallis were used to identify and compare categorical variables across agroecological zones. An index was used to assess the importance of each criterion. The majority of the farmers were males with secondary education, and socio-economic characteristics were non-significant (*P* > 0.05) across agroecological zones. Farmers bred their goats all year-round, with uncontrolled mating practices and used bucks born within the flock for breeding. The breeding season, purpose of keeping bucks and source of breeding bucks were statistically different (*P* < 0.05) across the agroecological zones. Most farmers culled goats based on age and practiced extensive system, with no significant difference in production systems across zones. Income generation was primary objective for keeping goats, and it varied significantly (*P* < 0.05) across the zones. Body size was the predominant selection criterion for both buck and does, with significant difference (*P* < 0.05) observed across the zones. These results are crucial for the designing of CBBP for Nguni goat farmers in Limpopo province, South Africa.

## Introduction

Goats are known as browsers and grazers that are highly selective and have means to survive and produce when there is shortage of feed (Asefa et al. 2015). Indigenous goats play a role to the lives of smallholder farmers at a rural level of South Africa since they are being used for cultural /religious purposes, food consumption and act as their bank (Mdladla et al. 2017; Jai Sunder et al. 2018). There are several indigenous goat ecotypes found in South Africa, which differs according to their body size, phenotypic characteristics, and geographic regions (Mtshali et al. 2021). Nguni goats are one of the indigenous goat ecotypes and are small to medium breed with multiple colours and their face profile is flat with dark pigmented muzzle (Snyman 2014). Indigenous goats are mostly kept by resource limited farmers, since they are easily managed, they can adapt to the harsh environmental conditions and have a short reproductive cycle (Tade et al. 2023). Indigenous goats drive the economy of farmers living in agroecological zones such as semi-arid and arid and contribute about 96% of meat and milk produced in developing countries (Mohammadabadi and Tohidinejad 2017; Mohammadabadi et al. 2021). Despite their contribution to the livelihood of smallholder farmers and their ability to thrive in harsh environments, their productivity is very low (Tilahun et al. 2023). This resulted in crossing these breeds with exotic breeds for their adaptability which then put the indigenous goats endangered (Marshall et al. 2019). This is due to the lack of clear strategies that can be employed to improve the production of the indigenous goats (Hagos et al. 2018). Therefore, understanding the breeding objectives, traits of preference and the production systems can assist in developing the community-based breeding program (CBBP), to improve productivity and conserve indigenous goats (Alebel et al. 2020). CBBP is a tool that can be employed by smallholder farmers to improve production of their goats (Mohammed et al. 2021). Studies have been conducted on documenting the breeding objectives, breeding practices and trait of preferences of goat farmers (Onzima et al. 2018; Tyasi et al. 2022) and, sheep farmers, where a slight variation in breeding objectives and trait of preferences were observed (Dossa et al. 2015). However, agroecological zones were not considered when studying goat farmers breeding objectives, breeding practices, and trait preferences for implementations of community-based breeding programme. Therefore, the objectives of this study were: 1) to identify the breeding objectives, breeding practices and traits preferences of Nguni goat farmers of Limpopo province, South Africa, and 2) to determine the effect of agroecological zones on Nguni goat farmers’ breeding objectives, breeding practices and trait preferences of Nguni goats’ farmers. This study will help farmers to improve the production and conservation of Indigenous Veld Goats.

## Materials and methods

### Study area

**Fig. 1:**
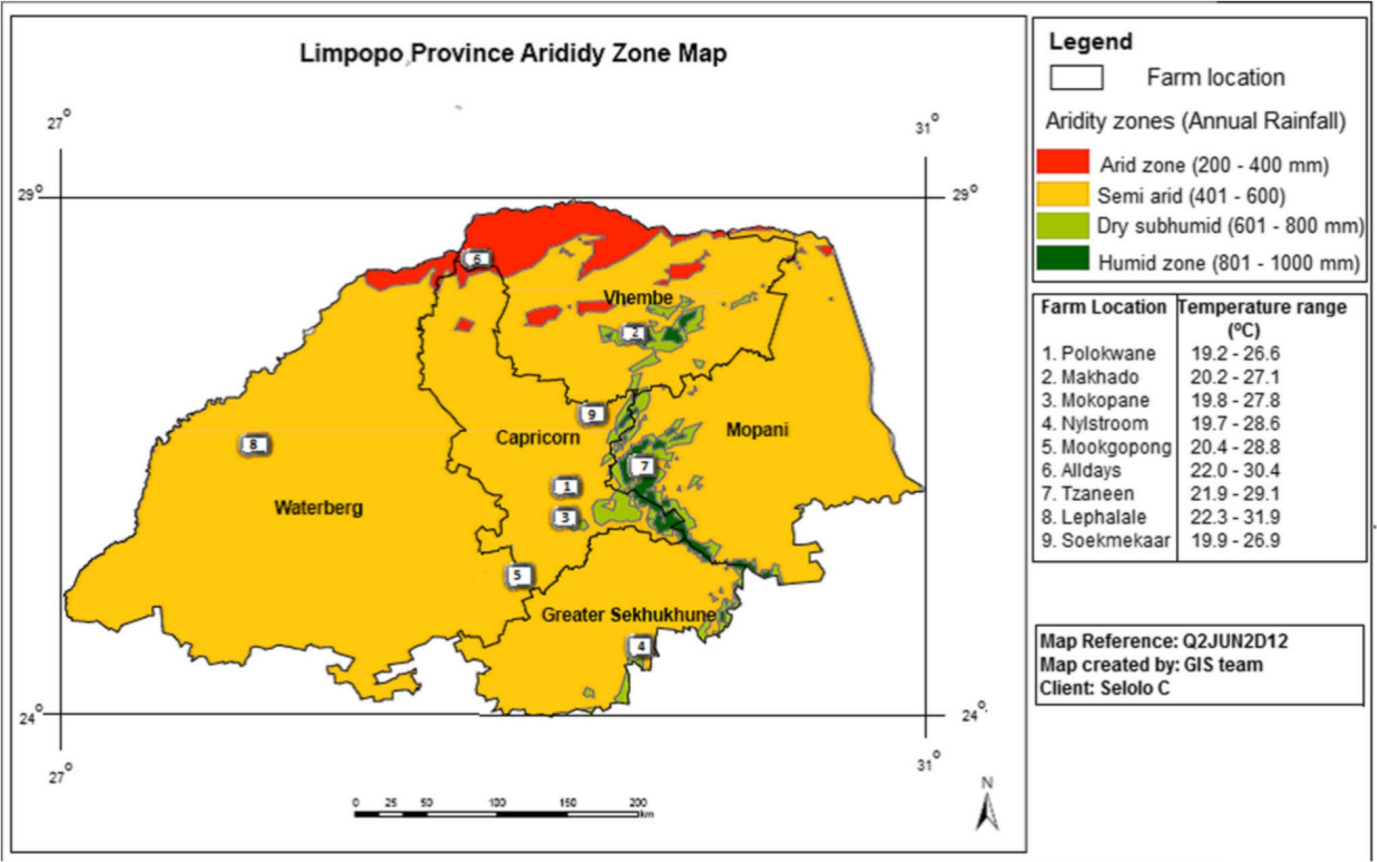
Limpopo map showing different agroecological zones (Mpofu et al. 2017)

The study was conducted in four districts of the Limpopo province, that correspond to four different agroecological zones (Fig. 1): semi-arid, humid, sub-humid, and arid. The agroecological zones from the province differ in terms of topography, rainfall, temperature, vegetation, and soil characteristics (Table 1) (Mpofu et al. 2017).

**Table 1:** Agroecological and their features in Limpopo province, South Africa

Agroecological zones	Veld types	Rainfall (annually)	Temperature (average)º
Arid	Sweet and mixed veld	Less than 254 mm	15 to 38 ºC
Semi-arid	Sour and mixed veld	Between 254 to 508 mm	25 to 35 ºC
Humid	Sour veld	Between 1500 to 2500 mm	24 to 27 ºC
Sub-humid	Sweet veld	Less than 1500 mm	27 to 35 ºC

Source: Mpofu et al. (2017)

### Study design

This study used a cross-sectional design, where data was gathered from each individual Nguni goat farmer once (Zangirolami-Raimundo et al. 2018). Farmers from each agroecological zone were visited once during data collection for interview using questionnaires.

### Sampling procedure

Four districts such as Vhembe, Capricorn, Mopani and Sekhukhune representing various agroecological zones (Arid, Semi-arid, Humid, and Sub-humid) within the Limpopo province were selected for the study owing to the presence of the IVGs Nguni goat producers. Out of 85 Nguni goat farmers in four districts of Limpopo province, a total of 33 farmers were selected based on their willingness to participate in the study. This was supported by Maswana et al. (2022), who indicated that a sample size of at least 26 farmers based on their willingness to participate in this kind of study is suitable.

### Data collection

A semi-structured questionnaire was utilized to examine the farmers preferred traits, breeding practices, production systems and production objectives. The questionnaire was pretested before the start of data collection to make sure the questions are appropriate for society and responses fall within the predicted ranges. The required modifications were implemented to ensure the questionnaires were ready. Researchers and trained enumerators conducted the interviews with farmers, allowing other member of the families to provide additional information in response to the questions posed. The data were collected in a participative manner, as explained by Tyasi et al. (2022). Farmers were free to provide any additional information not covered in the questionnaire.

### Data analysis

The Statistical Package for Social Sciences version 29.0 (IBM SPSS 2023) was employed for data analysis. Descriptive statistics such as frequencies and percentages were used for categorical variables including socio-economic characteristics, breeding practices and production systems. Chi-square statistics were used to compare the mentioned categorical variables between four agroecological zones. Reasons for culling, breeding objectives and selection criteria were calculated for the importance of each criterion and estimated by calculating the index for ranking as explained by Zewdu et al. (2018). The index used for all traits was: Index = sum (3 × rank1 + 2 × rank2 + 1 × rank3) for individual trait/sum (3 × rank1 + 2 × rank2 + 3 × rank1) for overall traits. The Kruskal–Wallis test was used to compare the means of reasons for culling, breeding objectives and selection criteria between four agroecological zones.

## Results

### Socio-economic characteristics

Table 2 displays socio-economics characteristics such as sex, marital status, age, and educational level. The results indicated that the majority of Nguni goat farmers from the four agroecological zones were males (77.10%) while the rest were females (22.90%). The majority of farmers in the four agroecological zones were married (69.50%), followed by single (25.90%) and widowed (4.60%). The majority of Nguni goat farmers were between 50–65 years (34.25%), followed by greater than 46 (28.20%), between 36—45 years (27.40%) and less than 35 years (10.30%). Most of the farmers had secondary educational level (45.10%), followed by those had tertiary education (43%) and the rest had primary education (6.80%). The findings indicated that the identified socio-economic characteristics were not significantly different (*P* > 0.05) between the four agroecological zones of Limpopo province, South Africa.

**Table 2:** Socio-economic status of Nguni goat farmers

Factor	Agroecological zones						
	Sub-humid N (%)	Semi-arid N (%)	Arid N (%)	Humid N (%)	Overall	Chi-square	*P*-value
Sex							
Males	7 (100.00%)	7 (87.50%)	7 (63.60%)	4 (57.10%)	(77.10%)		
Females	0 (0.00%)	1 (12.50%)	4 (36.40%)	3 (42.90%)	(22.90%)	5.04	0.17^ ns^
Marital status							
Single	2 (28.60%)	3 (37.50%)	1 (9.10%)	2 (28.60%)	(25.90%)		
Married	5 (71.40%)	5 (62.50%)	8 (72.70%)	5 (71.40%)	(69.50%)		
Widowed	0 (0.00%)	0 (0.00%)	2 (18.20%)	0 (0.00%)	(4.60%)	5.81	0.45^ ns^
Age							
< 35 years	2 (28.60%)	1 (12.50%)	0 (0.00%)	0 (0.00%)	(10.30%)		
36–45 years	2 (28.60%)	2 (25.00%)	3 (27.30%)	2 (28.60%)	(27.40%)		
> 46 years	1 (14.30%)	3 (37.50%)	2 (18.20%)	3 (42.90%)	(28.20%)		
50–65 years	2 (28.60%)	2 (25.00%)	6 (54.50%)	2 (28.60%)	(34.20%)	7.90	0.54^ ns^
Educational level							
No formal education	0 (0.00%)	0 (0.00%)	2 (18.20%)	0 (0.00%)	(4.60%)		
Primary	0 (0.00%)	0 (0.00%)	2 (18.20%)	1 (9.10%)	(6.80%)		
Secondary	3 (42.90%)	3 (37.50%)	6 (54.50%)	15 (45.50%)	(45.10%)		
Tertiary	4 (57.10%)	5 (62.50%)	1 (9.10%)	3 (42.90%)	(43.00%)	11.13	0.27^ ns^

^ns^: non-significant; ^*^: significant at (*P* < 0.05); ^**^: significant at (*P* < 0.01)

### Breeding practices

Table 3 shows the breeding practices such as breeding season, mating systems, methods of controlling mating, culling methods, breed type, purpose of breeding bucks, sources of breeding bucks and reasons for uncontrolled mating of Nguni goat farmers. The majority of goat farmers breed their goats all year round (57.70%), followed by winter (25.30%), autumn (7.20%) and summer (6.70%). Thus, there was a highly significant difference (*P* < 0.01) between four agroecological zones on breeding season. About 56.70% of the farmers practiced uncontrolled mating, and 35.40% used controlled mating. There was no significant difference (*P* > 0.05) between four agroecological zones, on mating system. On the ways of controlling mating, about 35.20% of farmers used culling and 35.10% farmers used castration. Lastly, 26.90% of farmers used both culling and castration. The results indicated that there was no significant difference (*P* > 0.05) between four agroecological zones, on the methods of controlling mating. The majority of farmers practiced selling (87.50%) as culling method, followed by slaughter (9.00%). There was no significant difference (*P* > 0.05) between four agroecological zones, on culling methods. The results indicated that all the farmers (100%) used Nguni goat ecotype. About 60.10% of farmers kept their bucks for mating only and about 33.60% of the farmers kept buck for both mating and fattening. There was a significant difference (*P* < 0.05) between four agroecological zones, on the purpose of keeping bucks. The majority of farmers used breeding bucks that were born from their flock (39.50%), followed by the purchased ones (35.30%) and the community (25.20%). There was a significant difference (*P* < 0.05) between four agroecological zones, on the sourcing of breeding bucks. Approximately 96.4% of farmers grazed their animals together and 3.60% of the farmers lacked awareness on the control mating of their animals. There was no significant difference (*P* > 0.05) between four agroecological zones on reasons for not controlling mating.

**Table 3:** Breeding practices of Nguni goat farmers

Breeding practices	Agroecological zones						
	Sub-humid	Semi-arid	Humid	Arid	Overall	Chi-square	*P*-value
Breeding Season							
Autumn	0 (0.00%)	0 (0.00%)	2 (28.60%)	0 (0.00%)	(7.20%)		
Spring	0 (0.00%)	1 (12.60%)	0 (0.00%)	0 (0.00%)	(3.20%)		
Winter	2 (28.60%)	0 (0.00%)	0 (0.00%)	8 (72.70%)	(25.30%)		
Summer	1 (14.30%)	1 (12.50%)	0 (0.00%)	0 (0.00%)	(6.70%)		
All year round	4 (57.10%)	6 (75.00%)	5 (71.40%)	3 (27.30%)	(57.70%)	26.55	0.001^**^
Mating systems							
Controlled	4 (57.10%)	3 (37.50%)	2 (28.60%)	2 (18.20%)	(35.40%)		
Partially controlled	0 (0.00%)	1 (12.50%)	1 (14.30%)	1 (9.10%)	(9.00%)		
Uncontrolled	3 (42.90%)	4 (54.10%)	4 (57.10%)	8 (72.70%)	(56.70%)	3.77	0.71^ ns^
Control mating							
Castration	2 (28.60%)	3 (37.50%)	2 (28.60%)	5 (45.50%)	(35.10%)		
Culling	3 (42.90%)	2 (25.00%)	3 (42.90%)	3 (27.30%)	(35.20%)		
Castration and culling	1 (14.30%)	3 (37.50%)	2 (28.60%)	3 (27.30%)	(26.90%)		
None	1 (14.30%)	0 (0.00%)	0 (0.00%)	0 (0.00%)	(3.60%)	5.61	0.78^ ns^
Culling methods							
Selling	5 (71.40%)	7 (87.50%)	7 (100.00%)	10 (90.90%)	(87.50%)		
Slaughter	1 (14.30%)	1 (12.50%)	0 (0.00%)	1 (9.10%)	(9.00%)		
Selling and slaughter	1 (14.30%)	0 (0.00%)	0 (0.00%)	0 (0.00%)	(3.60%)	5.01	0.54^ ns^
Breed type							
Nguni goats	7 (100.00%)	8 (100.00%)	7 (100.00%)	11 (100.00%)	(100.00%)		
Purpose of breeding bucks							
Mating	5 (71.40%)	2 (25.00%)	5 (71.40%)	8 (72.70%)	(60.10%)		
Fattening	0 (0.00%)	2 (25.00%)	0 (0.00%)	0 (0.00%)	(6.30%)		
Mating and fattening	2 (28.60%)	4 (50.00%)	2 (28.60%)	3 (27.30%)	(33.60%)	9.34	0.02^*^
Source of breeding buck							
Born from the flock	4 (57.10%)	1 (12.50%)	3 (42.90%)	5 (45.50%)	(39.50%)		
Community	0 (0.00%)	1 (12.50%)	3 (42.90%)	5 (45.50%)	(25.20%)		
Purchase	3 (42.90%)	6 (75.00%)	1 (14.30%)	1 (9.10%)	(35.30%)	13.72	0.03^*^
Reason for uncontrolled mating							
Grazing together	6 (85.70%)	8 (100.00%)	7 (100.00%)	11 (100.00%)	(96.40%)		
Lack of awareness	1 (14.30%)	0 (0.00%)	0 (0.00%)	0 (0.00%)	(3.60%)		
Both	0 (0.00%)	0 (0.00%)	0 (0.00%)	0 (0.00%)	(0.00%)	3.83	0.28^ ns^

^ns^: non-significant; ^*^: significant at (*P* < 0.05); ^**^: significant at (*P* < 0.01)

### Reasons for culling

Table 4 presents the Nguni goat farmers reasons for culling in four agroecological zones. The majority of farmers culled their goats based on old age (0.47), followed by undesired colour (0.30), undesired body conformation (0.29) disease resistance (0.25), and poor reproduction (0.24). Farmers reasons for culling breeding stocks differed within the agroecological zones. In the humid zone, old age (0.40) was the main reason for culling, followed by undesired body conformation (0.26) and undesired coat colour (0.14). Old age (0.47) was the main reason in semi-arid areas, followed by undesired body conformation (0.21) and coat colour (0.20). In sub-humid areas, old age (0.64) was the main reason for culling, followed by diseases resistance (0.43) and poor reproduction (0.38). Undesired coat colour (0.44) was the main reason for culling in arid zones, followed by poor reproduction (0.42) and diseases resistance (0.39).

**Table 4:** Reasons for culling breeding goats as ranked by Nguni goat farmers

Factors	Agroecological zones																
	Humid (*N* = 7)				Arid (*N* = 11)				Sub-humid (*N* = 7)				Semi-arid (*N* = 8)				
	R1	R2	R3	Index	R1	R2	R3	Index	R1	R2	R3	Index	R1	R2	R3	Index	Overall-Index
Undesired body confirmation	3	1	0	0.26	0	5	4	0.21	2	1	4	0.29	4	2	2	0.38	0.29
Old age	4	2	1	0.40	9	1	2	0.47	4	2	1	0.64	3	3	2	0.35	0.47
Diseases	0	0	4	0.10	1	0	2	0.08	4	3	0	0.43	4	3	1	0.39	0.25
Undesired coat colour	0	2	2	0.14	0	5	3	0.20	4	2	1	0.41	5	3	0	0.44	0.30
Poor reproduction	0	2	0	0.10	1	0	0	0.05	3	3	1	0.38	4	4	0	0.42	0.24

*R* Rank, *N* number of goat farmers, *N* sample size, *I* Index, *I* Sum of [3 for rank 1 + 2 for rank 2 + 1 for rank 3] given an individual reasons by sum [3 for rank 1 + 2 for rank 2 + 1 for rank 3] for all reasons

### Reasons for culling and their significant level

Table 5 shows the reasons for culling and their significance level across agroecological zones. The results showed that across four agroecological zones, undesired body confirmation and poor reproduction varied statistically at *P* < 0.05 and *P* < 0.01, respectively. The old age, disease resistance and undesired coat colour were similar (*P* > 0.05) across the agroecological zones.

**Table 5:** Mean ranks of reason for culling breeding stocks and their significant level according to Kruskall-Wallis test

Factor	Agroecological zones					
	Humid	Arid	Sub-humid	Semi-arid		
	Mean rank	Mean rank	Mean rank	Mean rank	Kruskall-Wallis test	Asymptotic significance
Undesired body conformation	7.57^b^	20.68^a^	21.50^a^	16.25^a^	10.632	0.014^*^
Old age	17.43	13.64	17.43	20.88	3.476	0.324^ ns^
Diseases resistance	19.43	11.18	19.00	21.13	6.675	0.083^ ns^
Undesired coat colour	15.79	18.23	15.71	17.50	0.473	0.925^ ns^
Poor reproduction	13.71^b^	9.05^b^	25.07^a^	23.75^a^	19.520	0.001^**^

^a,b^:superscript indicating difference between means; *, **:Significance at *P* < 0.05 and *P* < 0.01, respectively; ^ns^: Not significant

### Production systems

Table 6 displays the production systems, supplementary feeding, season of supplementing and types of feed. About 83.00% of farmers practised extensive production system and, about 9.80% and 7.20% of them practiced semi-intensive and intensive production systems, respectively. The majority (89.60%) of farmers supplemented their goats. About 78.00% of the farmers supplemented their goats in winter and about 22.00% supplemented all year round. About 60.60% of the farmers used maize as supplements, and about 39.40% of them used other feeds. The results indicated a significant difference (*P* < 0.05) between four agroecological zones on the types of feed used for supplementing. The results indicated that the agroecological zones were not significantly different (*P* > 0.05) for production systems, supplementary feeds, and season for supplementing.

**Table 6:** Production systems practiced by Nguni goat farmers

Factors	Agroecological zones						
	Sub-humid	Semi-arid	Humid	Arid	Overall	Chi-square	*P*-value
Production system							
Extensive	5 (71.40%)	6 (75.00%)	6 (85.70%)	11 (100.00%)	(83.00%)		
Semi-intensive	0 (0.00%)	2 (25.00%)	1 (14.30%)	0 (0.00%)	(9.80%)		
Intensive	2 (28.60%)	0 (0.00%)	0 (0.00%)	0 (0.00%)	(7.20%)	12.04	0.06^ ns^
Supplementary feeding							
Yes	7 (100.00%)	8 (100.00%)	6 (85.70%)	8 (72.70%)	(89.60%)		
No	0 (0.00%)	0 (0.00%)	1 (14.30%)	1 (14.30%)	(7.20%)	4.47	0.22^ ns^
Season of supplementing							
Winter	5 (71.40%)	7 (87.50%)	5 (71.40%)	9 (81.80%)	(78.00%)		
All year round	2 (28.60%)	1 (12.50%)	2 (28.60%)	2 (18.20%)	(22.00%)	0.88	0.83^ ns^
Type of feeds							
Maize	0 (0.00%)	6 (75.00%)	6 (85.70%)	9 (81.80%)	(60.60%)		
Other	7 (100.00%)	2 (25.00%)	1 (14.30%)	2 (18.20%)	(39.40%)	15.74	0.001^**^

^ns^: non-significant; ^*^: significant at (*P* < 0.05); ^**^: significant at (*P* < 0.01)

### Breeding objectives

The results of the breeding objectives (Table 7) indicated that the majority of farmers kept goats for income generation (0.44), followed by meat consumption (0.34) and savings (0.32). Farmers breeding objectives differed within the agroecological zones. Where meat consumption (0.44) was the major reason for keeping goats in semi-arid, followed by income generation (0.38) and savings (0.33). In sub-humid areas, income generation (0.48) was the major reason, followed by saving (0.38) and meat consumption (0.29).

**Table 7:** Breeding objectives as ranked by Nguni goat farmers

Factors	Agroecological zones																
	Humid (*N* = 7)				Arid (*N* = 11)				Sub-humid (*N* = 7)				Semi-arid (*N* = 8)				
	R1	R2	R3	Index	R1	R2	R3	Index	R1	R2	R3	Index	R1	R2	R3	Index	Overall-Index
Income	4	3	0	0.43	4	6	1	0.38	6	1	0	0.48	6	2	0	0.46	0.44
Meat	1	4	2	0.31	7	4	0	0.44	1	3	3	0.29	1	5	2	0.31	0.34
Ceremony	0	2	5	0.21	0	6	5	0.26	0	2	5	0.21	0	3	5	0.23	0.23
Savings	1	3	3	0.29	4	3	4	0.33	3	3	1	0.38	0	5	3	0.27	0.32
Manure	0	0	0	0.00	0	0	11	0.17	0	1	6	0.19	0	0	0	0.00	0.09
Wealth	0	0	7	0.17	0	2	9	0.20	0	0	0	0.00	0	0	0	0.00	0.10
Breeding	1	0	0	0.07	0	0	0	0	1	0	0	0.07	1	1	0	0.10	0.06

*R* Rank, *N* sample size, *I* Index, *I* Sum of [3 for rank 1 + 2 for rank 2 + 1 for rank 3] given an individual reasons by sum [3 for rank 1 + 2 for rank 2 + 1 for rank 3] for all reasons

### Breeding objectives and their significant level

Table 8 shows income, meat consumption, ceremony, savings, manure, wealth, and breeding with their significant level across agroecological zones. Manure and wealth differed statistically at *P* < 0.01, while meat consumption differed statistically at *P* < 0.05 across four agroecological zones. Income, ceremony, savings, and breeding didn’t differ statistically (*P* > 0.05) across agroecological zones.

**Table 8:** Mean ranks of farmers breeding objectives and their significant level according to Kruskall-Wallis test

Factor	Agroecological zones					
	Humid	Arid	Sub-humid	Semi-arid		
	Mean rank	Mean rank	Mean rank	Mean rank	Kruskall-Wallis test	Asymptotic significance
Income	17.36	21.27	12.79	14.50	5.510	0.138^ ns^
Meat	19.93^a^	10.23^b^	21.57^a^	19.75^a^	9.713	0.021^*^
Ceremony	18.79	14.50	18.79	17.31	1.703	0.636^ ns^
Savings	18.86	15.73	12.29	21.25	4.170	0.244^ ns^
Manure	8.00^b^	25.00^a^	23.71^a^	8.00^b^	31.036	0.001^**^
Wealth	25.50^a^	23.86^a^	8.00^b^	8.00^b^	30.211	0.001^**^
Breeding	17.29	15.00	17.29	19.25	2.855	0.415^ ns^

^a,b^:superscript indicating difference between means; *, **:Significance at *P* < 0.05 and *P* < 0.01, respectively; ^ns^: Not significant

### Selection criteria for breeding bucks

Table 9 indicates selection criteria for breeding bucks. The results indicated that when selecting breeding bucks, body size (0.45), was the first trait to be looked at, followed by disease resistance (0.41) and growth rate (0.35). Farmers trait of preferences differed within the agroecological zones. Disease resistance (0.60) was the first trait to be looked at when selecting breeding buck in humid zone, followed by body size (0.50) and scrotal circumference (0.29). In semi-arid zone, farmers looked at body size (0.47) first, followed by the appearance (0.38) and growth rate (0.30). Growth rate (0.45) was the first trait when selecting breeding buck in sub-humid, followed by disease resistance (0.40) and coat colour (0.38). Body size (0.46) was the first trait to looked at in arid zone, followed by disease resistance (0.38), growth rate (0.38) and coat colour (0.35).

**Table 9:** Selection criteria for breeding buck as ranked by Nguni goat farmers

Factors	Agroecological zones																
	Humid (*N* = 7)				Arid (*N* = 11)				Sub-humid (*N* = 7)				Semi-arid (*N* = 8)				
	R1	R2	R3	Index	R1	R2	R3	Index	R1	R2	R3	Index	R1	R2	R3	Index	Overall-Index
Appearance	0	0	0	0.00	4	6	1	0.38	1	2	4	0.26	1	3	4	0.27	0.23
Body size	7	0	0	0.50	9	2	0	0.47	2	4	1	0.36	6	2	0	0.46	0.45
Disease resistance	2	4	1	0.60	0	6	5	0.26	5	0	2	0.40	4	2	2	0.38	0.41
Scrotal circumference	0	5	2	0.29	0	6	5	0.26	0	3	4	0.24	0	6	2	0.29	0.27
Growth rate	0	4	3	0.26	2	5	4	0.30	5	2	0	0.45	4	2	2	0.38	0.35
Mating ability	0	0	7	0.17	0	3	8	0.21	4	0	3	0.36	2	3	3	0.27	0.25
Maternal history	0	1	1	0.07	0	0	4	0.06	0	0	0	0.00	0	0	1	0.02	0.01
Paternal history	0	0	0	0.00	0	0	0	0	0	0	0	0.00	0	0	1	0.02	0.01
Coat colour	0	3	4	0.24	0	4	7	0.23	2	5	0	0.38	3	3	2	0.35	0.30

*R* Rank, *N* number of goat farmers, *N* sample size, *I* Index, *I* Sum of [3 for rank 1 + 2 for rank 2 + 1 for rank 3] given an individual reasons by sum [3 for rank 1 + 2 for rank 2 + 1 for rank 3] for all reasons

### Selection criteria for breeding bucks and their significant level

The results of selection criteria for breeding bucks and their significant level (Table 10) indicates that appearance, coat colour varied statistically at *P* < 0.01 across agroecological zones. The body size, disease resistance and mating ability varied statistically at *P* < 0.05 across the agroecological zones. The results further indicated that the scrotal circumference, growth rate, maternal and paternal history didn’t vary statistically (*P* > 0.05) across the agroecological zones.

**Table 10:** Mean ranks of selection criteria for breeding bucks and their significant level according to Kruskall-Wallis test

Factor	Agroecological zones					
	Humid	Arid	Sub-humid	Semi-arid		
	Mean rank	Mean rank	Mean rank	Mean rank	Kruskall-Wallis test	Asymptotic significance
Appearance	4.50^b^	15.50^a^	24.21^a^	23.69^a^	21.18	0.001^**^
Body size	12.50^b^	15.41^b^	24.57^a^	16.50^b^	10.18	0.017^*^
Disease resistance	12.14^b^	23.32^a^	13.50^b^	15.63^ba^	8.60	0.035^*^
Scrotal circumference	15.21	18.00	19.93	14.63	2.06	0.55^ ns^
Growth rate	19.43	20.64	10.43	15.63	6.07	0.10^ ns^
Mating ability	23.00^a^	19.32^ba^	11.86^b^	13.06^b^	9.07	0.028^*^
Maternal history	15.43	19.68	15.93	15.63	2.53	0.470^ ns^
Paternal history	18.29	16.00	16.00	18.13	2.48	0.479^ ns^
Coat colour	21.00^ab^	21.91^ab^	10.14^b^	12.75^b^	10.80	0.013^**^

^a,b^:superscript indicating difference between means; *, **:Significance at *P* < 0.05 and *P* < 0.01, respectively; ^ns^: Not significant

### Selection criteria for breeding does

The results of the selection criteria for breeding does (Table 11) indicated that the first trait that the farmers looked into when selecting breeding does was body size (0.38), followed by growth rate (0.35) and milk production (0.34). Farmers trait of preferences differed within the agroecological zones. In semi-arid areas, growth rate (0.41) was the first trait to be considered when selecting breeding does, followed by litter size (0.39) and milk production (0.33). Litter size (0.43) was the first trait to be considered in sub-humid, followed by body size (0.40), disease resistance (0.40) and mothering ability (0.38). In arid zones, farmers looked into body size (0.44) first, followed by coat colour (0.40), appearance (0.38) and milk production (0.38). The agroecological zones had a non-significant difference (*P* > 0.05) on the selection criteria of farmers on breeding does.

**Table 11:** Selection criteria for breeding does as ranked by Nguni goat farmers

Factors	Agroecological zones																
	Humid (*N* = 7)				Arid (*N* = 11)				Sub-humid (*N* = 7)				Semi-arid (*N* = 8)				
	R1	R2	R3	Index	R1	R2	R3	Index	R1	R2	R3	Index	R1	R2	R3	Index	Overall-Index
Appearance	0	0	0	0.00	0	0	0	0.00	0	0	0	0	4	2	2	0.38	0.10
Body size	6	0	1	0.45	9	2	0	0.24	4	2	1	0.40	6	1	1	0.44	0.38
Diseases	1	3	3	0.24	0	2	9	0.20	4	2	1	0.40	2	2	4	0.29	0.28
Kidding interval	0	0	0	0.00	0	8	3	0.29	3	2	2	0.36	2	3	3	0.31	0.24
Growth rate	2	5	0	0.38	6	4	1	0.41	1	2	4	0.26	3	2	3	0.33	0.35
Paternal history	0	0	0	0.00	0	0	0	0.00	0	0	1	0.02	0	0	0	0	0.001
Maternal history	0	0	0	0.00	0	0	0	0.00	0	0	1	0.02	0	0	0	0	0.01
Mothering ability	1	6	0	0.36	0	2	9	0.20	3	3	1	0.38	2	4	2	0.33	0.32
Milk production	3	3	0	0.35	3	5	3	0.33	2	3	1	0.31	3	4	1	0.38	0.34
Fertility	0	0	0	0.00	0	0	0	0.00	0	0	0	0	0	0	0	0	0.00
Litter size	0	4	3	0.26	6	3	2	0.39	4	3	0	0.43	0	3	4	0.20	0.32
Coat colour	0	2	5	0.21	0	5	6	0.24	3	2	2	0.36	3	5	0	0.40	0.30

*R* Rank, *N* number of goat farmers, *N* sample size, *I* Index, *I* Sum of [3 for rank 1 + 2 for rank 2 + 1 for rank 3] given an individual reasons by sum [3 for rank 1 + 2 for rank 2 + 1 for rank 3] for all reasons

### Selection criteria for breeding does and their significant level

The results of the selection criteria for breeding does and their significant level (Table 12) indicated that the appearance, kidding interval and mothering ability varied statistically at *P* < 0.01 across the agroecological zones. The litter size and coat colour varied statistically at *P* < 0.05 across the agroecological zones. The results further indicated that the body size, disease resistance and milk production didn’t vary statistically (*P* > 0.05) across the agroecological zones.

**Table 12:** Mean ranks of selection criteria for breeding does and their significant level according to Kruskall-Wallis test

Factor	Agroecological zones					
	Humid	Arid	Sub-humid	Semi-arid		
	Mean rank	Mean rank	Mean rank	Mean rank	Kruskall-Wallis test	Asymptotic significance
Appearance	13.00^b^	13.00^b^	13.00^b^	29.50^a^	31.31	0.001^**^
Body size	17.00	15.86	17.00	18.56	0.92	0.819^ ns^
Disease resistance	14.71	21.36	14.71	15.00	4.31	0.230^ ns^
Kidding interval	7.50^b^	24.32^a^	7.50^b^	23.56^a^	26.66	0.001^**^
Growth rate	17.36	14.50	17.36	19.81	1.73	0.629^ ns^
Mothering ability	11.93^b^	25.36^a^	11.93^b^	14.38^b^	15.84	0.001^**^
Milk production	14.79	19.91	14.79	16.88	2.12	0.547^ ns^
Litter size	19.29^a^	10.50^b^	19.29^a^	21.94^a^	9.14	0.027^*^
Coat colour	21.21^a^	18.68^a^	21.21^a^	7.31^b^	13.61	0.003*

^a,b^:superscript indicating difference between means; *, **:Significance at *P* < 0.05 and *P* < 0.01, respectively; ^ns^: Not significant

## Discussion

Knowing the production objectives, breeding practices and trait preferences by involving farmers is very crucial in designing and implementing a viable breeding program at a rural level (Abebe et al. 2020).

### Socio-economic characterization

The current study identified the Nguni goat farmers socio-economic status, breeding objectives, trait preferences, breeding practices and their production systems. The socio-economic characteristics findings revealed that men were the most goat farmers in current study. The finding of the current study is consistent with the findings of Abd-Allah et al. (2019), where males were dominating gender in goat farming. Similarly, Alemu (2015), reported that men were the dominating goat keepers. This is due to the African culture, where men are regarded as the head of the house and expected to lead the livestock farming (Tyasi et al. 2022). However, Agholor et al. (2022), reported different results where females were found to be dominating in goat farming in Bushbuckridge South Africa. The study revealed that sex of the goat farmers was statistically similar across the agroecological zones. The majority of the goat farmers in the current study had a secondary and followed by tertiary educational level in the current study. The findings were in contrast with the findings of Praveena et al. (2014) and Tudu and Roy (2015), who reported that most of the goat farmers were not educated. However, the results of the current study were in accordance with the findings of Tyasi et al. (2022). Literate farmers can learn fast and think critically when the technical knowledge is needed (Islam et al. 2018). The findings suggest that working with literate farmers will assist in getting relevant information and will be easy to implement the breeding program. The findings of the study further indicated educational level were statistically similar across the agroecological zones.

### Breeding practices

The breeding practices findings of this study showed that Nguni goat farmers breed their animals all year round. The results of the current study were in contrast with the findings of Sharma and Khadse (2021), who reported that farmers from Akola and Hingoli district of Maharashtra India breed their animal in spring season. Similarly, Getaneh et al. (2022), reported that goat farmers from East of Gojjam zone and Amhara region of Ethiopia practice seasonal breeding. For farmers in the current study to breed all year round, might be that they are farming under communal land where all animals from different farmers graze together. The findings further indicated that breeding seasons differed significantly across the four agroecological zones. Most of the farmers in the current study practiced uncontrolled mating system. This was due to scarcity of land, thus animals from different farmers grazed together. The current study findings were in accordance with the findings of Zergaw (2014) and Getaneh et al. (2022) who reported that majority of farmers in Ethiopia practiced uncontrolled mating. This might be due to the extensive production system practiced, where goats from various farmers graze and browse together. However, the mating system were statistically similar across four agroecological zones. Nguni goat farmers controlled the mating through culling, where they were selling those that, are not fit for breeding. The findings were in accordance with the findings of Maswana et al. (2022), who reported that non-descript indigenous farmers from Madiga village of South Africa performed culling as the mating control method in their area. Findings of Muluneh and Awoke (2021) were in contrast with the findings of the study and revealed that farmers from West Guji zone of Ethiopia castrated their animals. Mating controls and culling methods were statistically similar across the studied agroecological zones. Most of the farmers kept the bucks for mating. The findings of Muluneh et al. (2016) were in contrast with current study, where it was found that majority of farmers kept their bucks for fattening. However, findings of Asefa et al. (2015) were in accordance with the findings of the study. This showed that the Nguni goat farmers focus on reproducing the animals so that they can be able to sell. The current study findings further revealed that purposes of keeping breeding bucks differed statistically across the agroecological zones. Farmers used breeding bucks born in the flock. Gebreyesus et al. (2013), also reported that most farmers use bucks that were born from the flock for breeding. Similarly, Nguluma et al. (2020), reported that indigenous goat farmers of Northern Tanzania used breeding bucks that were born in the flock. This clearly indicate that there is high inbreeding occurring in the flock of the farmers from current study.

On the reasons of culling, the current study revealed that farmers from Lepelle-Nkumpi Local Municipality of Limpopo province South Africa culled their goats based on old age. Tyasi et al. (2022), also reported that goat farmers culled their animals based on old age. Findings of Nguluma et al. (2020), were in contrast with the findings of the study, where goat farmers from North Tanzania culled their goats-based body condition score. Similarly, the findings of Badjibassa et al. (2024), were in contrast with the findings of the study where goat farmers from Burkina Faso culled their goats based on undesired body conformation. These means that Nguni goat farmers preferred to farm with young animals. The current study indicated that undesired body conformation and poor reproduction differed significantly across four agroecological zones. Whereas old age, disease resistance and undesired coat colour were statistically similar across four agroecological zones.

### Production system

On the production system, the current study revealed that most of the Nguni goat farmers raised their goats under extensive production system where majority of the farmers do supplement their goats. The findings were in contrast with the findings of Yakubu and Achapu (2017), who reported that goat farmers in central of Nigeria kept their goats under semi-intensive production system. The findings were in accordance with findings of Yemane et al. (2022), who reported that indigenous goat farmers in Southwestern Ethiopia their goat’s dependent much on the communal land for grazing and browsing. This means that most of the Nguni goat farmers are poor and coming from rural areas where there is not enough space for farming. The current study further revealed that farming systems of the Nguni goat farmers were statistically similar across the agroecological zones.

### Breeding objectives

A viable breeding program requires one to know the farmers’ breeding objectives since such information is vital in defining breeding goals important characteristics which might have impact on the motivation and profitability of long-term breeding program (Nguluma et al. 2020). On the breeding objectives, the current study revealed that majority of the farmers kept their goats for generating of income that help them when there is an emergency, since goats can perform and produce in uncoverable conditions. Similarly, Tolossa and Benerje (2022) reported that Arsi-bale goat farmers of Ethiopia kept their goats for income. Similarly, Abraham et al. (2017), Tegegn and Askale (2017), Nguluma et al. (2020), Yemane et al. (2022), reported that goat farmers from Ethiopia kept their for-income generation. This is based on farmers’ need for money to pay fees and run other household errands and agricultural inputs. Conversely, Gebre et al. (2023) reported that milk production was the primary objective of farmers to keep goats in Afar region, Ethiopia. This indicates that farmers from Afar region, Ethiopia were more aware about the nutritional values of the milk, unlike the farmers from four agroecological zones of Limpopo province. In contrast, Tesfahun et al. (2017) indicated that the primary purpose of keeping goats was social prestige in Benatsemay and Hamer districts in Southern Ethiopia. These are those farmers with the mentality of having many livestock comes with the respect from society and shows that you are rich. Similarly, Gebre et al. (2020), reported that goat farmers production objective was milk production in Aba’ala, Afar region of Ethiopia. Tyasi et al. (2022), reported similar findings with the current study where goat farmers from Lepelle-Nkumpi Local Municipality of South Africa kept their goats for income generation. Furthermore, the findings revealed that meat consumption, manure and wealth differed statistically across four agroecological zones. This suggest that farmers from different agroecological zones had different reasons for breeding their animals in the study.

### Selection criteria of breeding bucks

Selection of good breeding buck is a crucial factor in livestock production that leads to improvement of on-going genetic improvement that link with the breeding objectives of the farmers (Ahmed 2017). Nguni farmers across the four agroecological zones selected their breeding bucks based on body size. Conversely, Assefa et al. (2023) reported that the primary trait for selecting breeding buck was coat colour in the northeastern Ethiopia. Similarly, the findings were in contrast with the findings of Yousuf et al. (2020), who reported that main trait to be considered when selecting breeding bucks was appearance. The current study findings were in accordance with the findings of Mtshali et al. (2021) and Tyasi et al. (2022), who reported that goat farmers primary selection criteria were body size in South Africa. However, Nguluma et al. (2020) reported that coat colour was the primary trait to be considered when selecting breeding buck. Body size does positively affect the selling price of the animals, hence the farmers in the current study considered it as a primary selection criterion. The results of the current study further revealed that appearance, body size, disease resistance, mating ability and coat colour differed statistically across agroecological zones.

### Selection criteria of breeding doe

The majority of Nguni goat farmers selected their breeding does based on body size. In contrast, Tabbaa et al. (2023) found that growth rate was the primary trait when selecting breeding does. Kebede and Usman (2023), reported that body conformation was the first trait to be considered when selecting breeding doe across agroecological zone. Conversely, Gebre et al. (2020) reported that goat farmers from pastoral and agro pastoral considered milk as the primary selection criterion. Similarly, Seid et al. (2015) reported that goat farmers from Wollega zone of western Ethiopia preferred litter size first when selecting their goats for breeding. The findings were in contrast with the findings of Gebreyowhens and Kumar (2018), who reported that Maefur goat farmers selected their breeding doe primarily on the growth rate. The findings of Mtshali et al. (2021), reported similar findings to the current study where body size was the primary selection criteria for indigenous goat farmers from North-West province of South Africa. Nguni goat farmers kept their goats for profit since their selection criteria is associated with the pricing of the animal. Further, appearance, kidding interval, mothering ability, litter size and coat colour differed statistically across four agroecological zones. It means that farmers from different agroecological zones had different selection criteria for breeding does. It further showed that body size, growth rate and milk production were statistically similar across agroecological zones.

## Conclusion

In conclusion, there were no differences across the studied agroecological zones in terms of gender of the farmers and educational levels. Most of the farmers were males and had a secondary educational level. The was a variation in breeding seasons, purpose of keeping bucks, source of breeding bucks and reasons for culling across the agroecological zones. The study further revealed a variation in some of the breeding objectives of the goat farmers across the agroecological zones and concluded that majority kept goats for income generation. On the selection criteria, the study concluded that there was a variation on some of the selection criteria of the goat farmers, and majority focused on body size as their primary trait in both breeding bucks and does. The breeding objectives, trait preferences and breeding practices identified in the current study can be used to design and implement a sustainable community-based breeding program of the Nguni goat farmers in four agroecological zones of Limpopo province South Africa.

## Data Availability

All data generated or analysed during this study are included in the manuscript.
